# Adenoid Cystic Carcinoma of Child: A Rare Case

**DOI:** 10.5005/jp-journals-10005-1266

**Published:** 2015-02-09

**Authors:** Meera Mathai, J Eugenia Sherubin, PG Agnihotri, GS Sangeetha

**Affiliations:** Postgraduate Student, Department of Oral Medicine and Radiology, Sree Mookambika Institute of Dental Sciences, Kanyakumari, Tamil Nadu, India; Reader, Department of Oral Medicine and Radiology, Sree Mookambika Institute of Dental Sciences, Kanyakumari, Tamil Nadu, India; Professor and Head, Department of Oral Medicine and Radiology, Sree Mookambika Institute of Dental Sciences, Kanyakumari, Tamil Nadu, India; Senior Lecturer, Department of Oral Medicine and Radiology, Sree Mookambika Institute of Dental Sciences, Kanyakumari, Tamil Nadu, India

**Keywords:** Adenoid cytic carcinoma, Child, Maxilla.

## Abstract

Adenoid cystic carcinoma (ACC) is the second most common malignant tumor affecting both major and minor salivary glands. Clinically, it is a slowly growing tumor with high propensity for local invasion, recurrence and distant metastasis. It is predominantly seen in the ffith and sixth decades of life. Here, we report a rare case of ACC affecting the right maxilla of a 12-year-old girl.

**How to cite this article:** Mathai M, Sherubin JE, Agnihotri PG, Sangeetha GS. Adenoid Cystic Carcinoma of Child: A Rare Case. Int J Clin Pediatr Dent 2014;7(3):206-208.

## INTRODUCTION

Adenoid cystic carcinoma (ACC) was first described by Robin et al in 1853. In 1859, Billroth suggested an alternate nomenclature ‘cylindroma’, for its cribriform appearance formed by tumor cells around cylindrical pseudolumina.^1^ However, the term ACC which is widely accepted and currently in use was suggested by Spies in 1930.^2^ Adenoid cystic carcinoma accounts for 22% of all salivary gland malignancies and is one of the most common malignant tumors of the minor salivary glands with palate being the most common site.^2,3^ It is predominantly seen among women in ffith and sixth decade of life.^2,3^ Adenoid cystic carcinoma is well known for its prolonged clinical course and tendency for delayed onset of distant metastases. The tumor has a well-described histopathologic appearance with certain features that may predict its prognosis.^3^ Treatment of these tumors includes: surgical excision and postoperative radiation. The role of chemotherapy for metastatic ACC is still controversial.^4,7^

## CASE REPORT

**Fig. 1:**
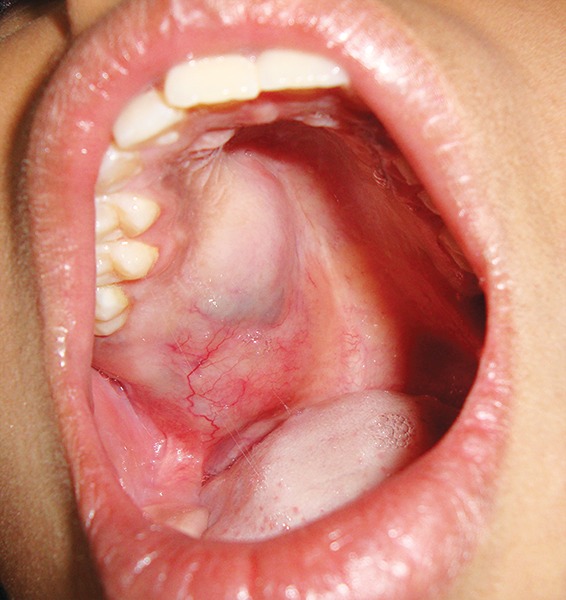
Intraoral photograph of swelling

**Fig. 2:**
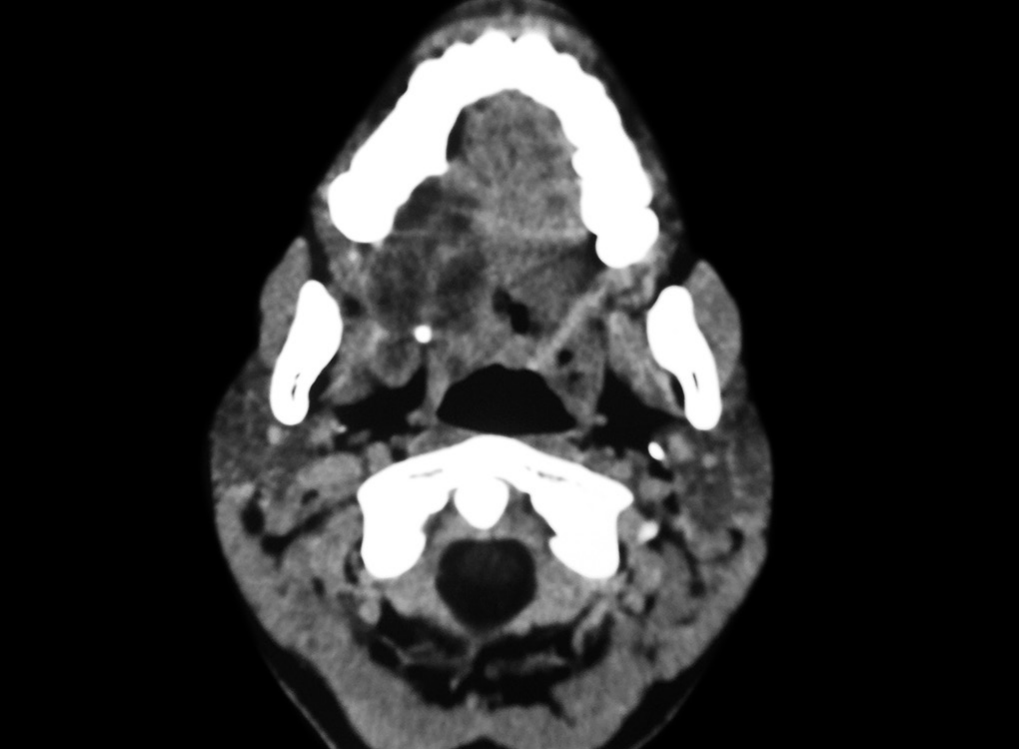
Computed tomography image of the lesion

A 12-year-old girl was referred to our department of oral medicine and radiodiagnosis for the evaluation of a swelling in the right posterior maxilla since 2 months. The swelling was initially asymptomatic and eventually developed dull and intermittent pain. Intraoral examination revealed a well-defined swelling of size 5 × 4 cm in the right posterior maxilla, extending anteriorly up to the distal aspect of 13, posteriorly covering the hamular notch and medially involving the soft palate (Fig. 1). The tooth 17 in relation to the swelling was found to be missing. The mucosa over the swelling appeared smooth and bluish in the anterior aspect and erythematous over the posterior aspect. On palpation, the swelling was tender and rubbery in consistency. There were no palpable regional lymph nodes. Patient's medical history was unremarkable with all biochemical examination results within normal limits. Considering the age and site, we made a clinical diagnosis of mucoepidermoid carcinoma of right maxilla with a differential diagnosis of ACC. The patient was subjected for radiographic examination (Fig. 2). Computed tomography (CT) showed a well-demarcated lobulated minimally enhancing soft-tissue density of mass 4.5 × 2.7 × 2.2 cm in the posterior half of right hard palate with thick enhancing septations causing scalloping and thinning of palatine bone-favoring a diagnosis of malignant salivary gland tumor.

**Fig. 3:**
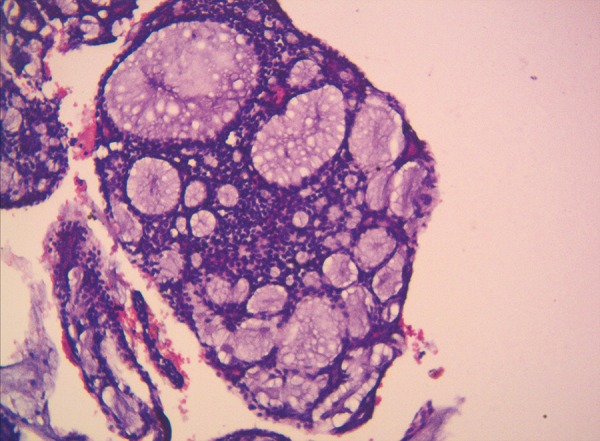
Histopathological slide

The histopathological examination of the specimen subjected after an incisional biopsy showed cribriform or Swiss Cheese pattern of tumor cells and basaloid epithelial cells rendering a fnal diagnosis of ACC (Fig. 3). Following which, the patient was subjected for a whole body CT screening to rule out metastasis and magnetic resonance imaging (MRI) head and neck to rule out perineural invasion.

**Fig. 4:**
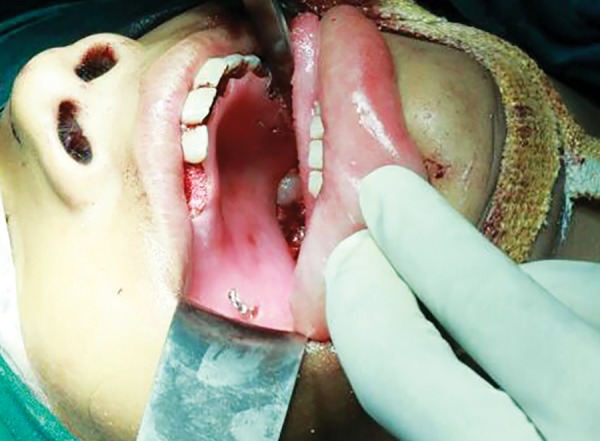
Stent placed after resection

The patient was treated with subtotal maxillectomy of right side followed by placement of surgical stent with surgical site packed with ribbon gauze impregnated with iodoform and glycerin (Fig. 4). Patient was reviewed after 2 weeks, patient made an unremarkable recovery. Patient is under our follow-up.

## DISCUSSION

Tumors of salivary glands are rare in children and malignant tumors are even more so^5^. Although ACC was thought to be mainly a disease of the elderly, a recent review reports an age range of 10 to 96 years. According to the gender predilection, even though few authors, such as Evesson and Cawson found a female predilection, majority of researchers in the literature have reported an equal distribution in both genders.^1^ Regarding the etiology, there is neither a universally accepted predisposing factors nor a family tendency for the development of ACC.^6^ Clinically, ACC always has an initial period of asymptomatic slow and indolent growth, where majority of the tumor goes unnoticed until it invades the local nerves and structures causing a delay in seeking treatment. Hence, most patients present with locally invasive disease at the initial visit itself. Even though this tumor grows slowly it widely infltrates with a characteristic mode of spread. It has a tendency for perineural spread accounting for pain in about 50% of cases.^3^ The propensity of this tumor to invade bone and spread along the base of the skull results in extensive intracranial invasion and involvement of the cranial nerves . Extensive intracranial invasion is the major cause of death in these tumors .^4^

Radiological investigations, especially CT is important to delineate the tumor, to plan extent of surgery and to look out for recurrences as a follow-up postoperatively. Pulmonary and skeletal surveys are important to rule out distant metastasis.^4^ Regarding metastasis of ACC, lymphatic spread is rare. In longstanding cases, distant metastasis occurs via bloodstream to lungs and bones. Most cases of metastatic ACC remain asymptomatic for a long time. Distant metastasis can appear years or even decades after the initial diagnosis.

Final diagnosis of ACC is primarily based on its characteristic histological features which play a significant role not only in diagnosing the tumor but also in determining treatment and prognosis. Among the three forms, cribriform, tubular and solid, solid subtype is considered to be the most aggressive tumor.^4^

Treatment of these tumors includes: surgical excision and postoperative radiation. The role of chemotherapy for metastatic ACC is still controversial.^4^ The clinical course of the disease is heterogeneous with some patients surviving decades and others surviving only months.^3^ The prognosis depends on factors like solid histological type, perineural spread, distant metastasis and recurrent local lesions.^8,9^ The microinvasion of bones, which is clinically and radiologically undetectable, frequently results in local recurrences even after surgery.^4^ Hence, a close surveillance and a long-term follow-up is mandatory in the management of this disease.

## CONCLUSION

Adenoid cystic carcinoma is one of the most biologically destructive and unpredictable tumor of the minor salivary glands occurring predominantly in the ffith and sixth decades of life. Its occurrence in children is extremely rare. Hence, an awareness among the dental fraternity about the existence of this disease in child population can ensure a better survival period for these patients and enhance their quality of life by our early diagnosis and interventions.
